# Evolution of Food Safety Features and Volatile Profile in White Sturgeon Caviar Treated with Different Formulations of Salt and Preservatives during a Long-Term Storage Time

**DOI:** 10.3390/foods10040850

**Published:** 2021-04-14

**Authors:** Annalaura Lopez, Federica Bellagamba, Erica Tirloni, Mauro Vasconi, Simone Stella, Cristian Bernardi, Mario Pazzaglia, Vittorio Maria Moretti

**Affiliations:** 1 Department of Veterinary Medicine, University of Milan, 26900 Lodi, Italy; federica.bellagamba@unimi.it (F.B.); mauro.vasconi@unimi.it (M.V.); vittorio.moretti@unimi.it (V.M.M.); 2 Department of Health, Animal Science and Food Safety, University of Milan, 26900 Lodi, Italy; erica.tirloni@unimi.it (E.T.); simone.stella@unimi.it (S.S.); cristian.bernardi@unimi.it (C.B.); 3 Agroittica Lombarda S.p.A., 25012 Calvisano, Italy; mario.pazzaglia@agroittica.it

**Keywords:** caviar, sturgeon, *Acipenser transmontanus*, food safety, volatile compounds, flavor

## Abstract

Caviar is a semi-preserved fish preparation in which cold storage (around 0 °C) and packaging under anaerobic conditions are fundamental to guarantee adequate safety parameters. Consumers seem to prefer caviar prepared with food salt only, but according to the needs of the different distribution channels, some preservatives are used in order to prolong its shelf life and to allow less restrictive storage conditions. Traditionally, the most common preservative was sodium tetraborate (borax), a salt that contributes to the sensory profile of caviar. However, due to its toxicity, borax has been banned in many countries, and the current trend is to reduce or eliminate its use. In this study, we evaluated the evolution of food safety parameters (pH, water activity, microbiological parameters) and the volatile profile during 14 months of storage in caviar samples treated with three different preservatives: I. exclusively NaCl, II. a mixture of borax and NaCl, and III. a mixture of organic acids and salts. Microbial presence was studied by means of plate counts; volatile organic compounds were identified on the sample headspace by means of solid phase microextraction with gas-chromatography and mass spectrometry. Results showed relevant differences among the three treatments investigated, with salt samples characterized by the highest viable counts and the greatest presence of volatile products driven by oxidative and spoilage processes, mainly occurring toward lipid and amino acids. On the contrary, the mixture of organic acids and salts showed the best response during the entire storage period. Finally, the employment of a multiparametric statistic model allowed the identification of different clusters based on the time of ripening and the preservative treatments used.

## 1. Introduction

Nowadays, caviar is a luxury food product distributed and appreciated worldwide. In the early 1700s, caviar was spread mainly in Caspian Countries, but during the 18th century, it started to be imported and cherished in Europe as well. Here it reached a “fashion status” among the wealthy class. During the last part of the 20th century, since the advantageous economical gain, caviar manufacturers started to practice overfishing toward wild sturgeon, and to massively produce caviar addressed to the export market [1]. This happened in any water basin representing the sturgeon’s natural habitat, both in the Eurasian and American countries. Natural stocks of sturgeons started to be heavily threatened by both anthropic alteration of the natural environment, pollution, and overfishing. Thus, in 2006 production of caviar coming from wild fisheries was banned by international law [2]. Consequently, sturgeon aquaculture started to notice a remarkable growth for the first fifteen years of the 21st century. According to the latest estimates in 2017, Italy was the 7th producer worldwide in terms of sturgeon biomass production (850 tonnes), and the 3rd in terms of aquaculture caviar production (43 tonnes) [3].

Caviar can be counted as a semi-preserved fish product [4]. Following the guidelines of the Codex Alimentarius Committee, within the European Union only the salt-treated eggs harvested from the Acipenseridae fish family specimens can be named as “caviar” [5]. This kind of product is characterized by values of NaCl in water phase > 6% (*w*/*w*) or a pH < 5.0. Chill storage (<5 °C) for these products is essential, and, together with the employment of anaerobic packaging conditions, it represents the main preventive measure against the hazardous growth of pathogenic microorganisms, such as *C. botulinum*. Failing that, the mesophilic and salt-resistant pathogens would be able to grow, representing a great risk to the consumers, since semi-preserved products are eaten without cooking [4]. Actually, even if caviar is produced under the highest hygiene conditions, many microbial contaminations may occur because of the high protein and lipid content and the lack of pasteurization steps [6]. The caviar product more appreciated by consumers is represented by sturgeon eggs prepared with food salt only [7]. Salt facilitates the preservation and brings out the exalted flavor. In 2006, Gussoni et al. [8] showed evidence of a higher lipid mobility in salted sturgeon eggs compared to unsalted eggs by measuring the relaxation decay of lipid components through nuclear magnetic techniques. Since caviar that is only salted is not treated with any preservative, it is mandatory for it to be stored at −2 or −3 °C.

However, salt alone is generally considered insufficient to preserve a high quality of caviar for a long term (over 6–8 months, depending on the concentration) [9]. Thus, according to the needs of the different distribution channels, in order to extend the shelf-life of caviar and to allow the mildest storage conditions, sturgeon eggs can be treated with some food preservatives. Particularly, the Commission Regulation (EU) No 1129/2011 [10] requires that within the European Union (EU), sturgeon caviar is the only food product that can be treated with boron as a food additive, in the form of boric acid (E284) and sodium tetraborate (borax–E285). The treatment of sturgeon eggs with boric acid and borax allows the conservation of caviar even at higher temperatures (2 and 4 °C) [9]. Boric acid and its salts are not considered suitable for use as food additives in many countries. However, in 2013, the European Food Safety Authority (EFSA) stated that boric acid and its salts are toxicologically acceptable for use as a preservative in genuine caviar. As a matter of fact, the EFSA evaluated that it is unlikely that consumers’ exposure to boron through caviar consumption occurs on a regular basis, and, thus, unlikely that a regular exceedance of the ADI, corresponding to 0.16 mg boron equivalent/kg bw/day, would occur [11].

Organic acids and their salts are microbial preservatives, frequently employed to inhibit the growth of microorganisms in food. They are well known to inhibit the growth of both bacterial and fungal cells by mean of several action mechanisms: membrane disruption, inhibition of essential metabolic activity, arousal of intracellular pH homeostasis stress, and so on. Particularly, sorbic acid (E200) and its salts, such as potassium sorbate (E202) are known to inhibit also the germination and outgrowth of bacterial spores [12]. Furthermore, many organic acids, such as ascorbic and isoascorbic acid (E300 and E315) are known as powerful antioxidants. They work as oxygen radical scavengers, allowing the reduction of the peroxyl radicals that propagate lipid peroxidation, known as the main mechanism by which fats and fat-containing foods undergo spoilage [13]. Within the EU, the usage of sorbic acid (E200), isoascorbic acid (E315), and their salts as antimicrobial and antioxidant preservatives is approved in a wide range of foods, included many preserved and semi-preserved fish products, such as fish roes (Annex II, [10]). 

Beyond the aspects of food safety, the concept of excellence for caviar is strictly linked to its flavor properties. Sensory evaluation is a fundamental step in the caviar production chain, and nowadays, the caviar market is defined by a wide diversification, linked to the availability of several products, each one characterized by different quality and different prices. High-quality caviar is undeniably characterized by the absence of off-flavors; samples that do not meet this requirement cannot be addressed to human consumption, as stated by the Codex Alimentarius standard [5], and represent a waste, and consequently an economic loss, for producers. It is known that the presence of off-flavors in caviar is mainly related to microbial contamination of the product [14], to the presence of metabolites driven by enzymatic reactions occurring under both aerobic and anaerobic conditions [15], and to the presence of lipophilic compounds absorbed by fish eggs during the farming stage [16]. Incorrect procedures occurring during the product preparation could lead to unacceptable deficiencies. For example, incorrect handling, bad storage temperature control, or incorrect manipulation of cans during the canning and re-canning steps could lead to contamination of the matrix or to the undesired presence of oxygen in cans, leading to product oxidation and deterioration.

So far, the influence of additives on the stability of caviar and its volatile profile has not been described in any previous study. Therefore, the aims of this work were: (a) to evaluate the impact of the use of three different additives on the development of organic volatile compounds in white sturgeon (*Acipenser transmontanus*) caviar during an extended storage time; (b) to assess the overall safety properties of the product under these conditions; and (c) to establish, if possible, which is the ideal ripening period for the marketing of caviars with optimal organoleptic and safety properties.

## 2. Materials and Methods

### 2.1. Sampling

Caviar samples were provided by an Italian caviar company (Agroittica Lombarda SpA, Calvisano, BS, Italy). Raw eggs were obtained from 12 sexually mature females of white sturgeon (*Acipenser transmontanus*). Roes were sieved and washed before the addition of the salt/preservative mixtures. Samples were treated following different formulas, added under aseptic conditions at the amount (% by weight) suggested by the good manufacturing practice rules which codify caviar production in the enterprise that supported the investigation—always complying, however, with the limit levels established and defined in the Commission Regulation (EU) No 1129/2011 [10] for fish roes and caviar (section 09.3)—as follows:

- NC series: sodium chloride (NaCl) was added at 3.8% by weight;
- Experimental OAM (organic acids mixture) series: an experimental preservative, made of a mixture of sodium chloride, sorbic acid (E200), potassium sorbate (E202), and isoascorbic acid (E315), was added at 4% by weight;
- BSM (borax and salt mixture) series: salt and sodium tetraborate (E285) mixture was added at 3.8% by weight.

Each sample was placed in a special metal can called the “original tin” containing 500 g or 1800 g of caviar. This type of tin helps the air and any excess liquid escape. Then, cans were kept at a storage temperature of −2 °C and handled according to the company’s processing protocol. Samples were collected and analyzed starting from the day of extraction (t0), and then at three different time points during the ripening, corresponding to 4 months (t1), 8 months (t2), and 14 months (t3).

The samples collection design is briefly reported in Table 1.

Samples were sent to university laboratories under refrigeration conditions and kept at the storage temperature until the analysis. Unfortunately, an exception was represented by BSM samples at t0, which were stored in freezing conditions before the chromatographic analysis because of technical problems that unexpectedly occurred with the analytical equipment.

### 2.2. PH and a_w_

The pH was measured at each sampling time using a pH meter (Amel Instruments, Milan, Italy), using 4.0 and 7.0 as calibration points. Water activity (a_w_) was determined using an Aw-DIO Hygromer humidity sensor (Rotronic, Bassersdorf, Switzerland), using 0.8 as the calibration point.

### 2.3. Microbial Counts

For microbial counts, 10 g of product were 10-fold diluted in chilled sterile diluent solution (0.85% NaCl and 0.1% peptone) and homogenized for 60 s in a Stomacher 400 (Seward Medical, London, UK). Appropriate 10-fold dilutions of the homogenates were prepared in chilled saline solution. Total mesophilic viable count (TVC) was determined onto plate count agar (PCA) (ISO 4833-1:2013 [17]).

### 2.4. Volatile Organic Compounds Profile

For each sample, an aliquot of 5 g of caviar was employed in the analysis without any treatment before volatile organic compounds (VOCs) extraction; each sample was analyzed in duplicate. VOCs were extracted by means of the solid phase microextraction (SPME) technique employed on the sample headspace (HS); then, analytes were separated and identified by gas chromatography coupled to mass spectrometry (GC-MS). The analytical conditions (SPME, GC, and MS parameters) were set according to the optimization used in Lopez et al. [18]. Briefly, DVB/CAR/PDMS 1 cm SPME fibers (Supelco) were used for the HS extraction, performed for 30 min at 60 °C. Then, VOCs were recovered by thermal desorption in the S/SL injection port of the GC system (6890 N Network GC system by Agilent Technologies) set at 250 °C, in splitless mode. The chromatographic separation was performed by an optimized oven temperature program (from 35 °C to 150 °C at 5 °C/min and then to 260 °C at 10 °C/min) employing a DB-5 MS column (30 m × 0.25 mm id, 0.25 um film thickness, Agilent Technologies). Helium was used as carrier gas, at a flow of 1 mL min^−1^ (pressure 6.71 psi). A 5973 Network Mass Selective Detector (Agilent Technologies, Inc., Santa Clara, CA, USA) was employed for VOCs detection and identification, working in EI mode (70 eV, scan range m/z 35–300 amu, scanning rate 5.19 scans sec^−1^). Data were acquired by Enhanced ChemStation software (Agilent Technologies, Inc., Santa Clara, CA, USA). A first tentative identification of key aroma compounds was performed by comparisons with mass spectra of compounds found in library data (NIST MS library). Then, the identification was performed by means of two approaches:

1. Identification by matching with van den Dool and Kratz [19] retention indices, calculated on the basis of a homologous series of n-alkane injected in the same analytical conditions of samples [20];
2. Identification by mass spectra of authentic standards injected in the same analytical conditions, when available. When analyzing the chemical standards, the injection port of the GC system was set in the split mode (split ratio 1:100) and 1 µL was injected. A purge flow of 50 mL/min was set at 2 min to avoid an oversaturation of the MS ion source.

### 2.5. Data Analysis

Chromatographic signals were normalized for each sample weight, and noise was subtracted for each run. Unmatched chromatographic peaks were heightened to values close to zero. Both chromatographic peak areas and microbial counts were log-transformed before performing a data-fusion process. First, a multifactorial model was built in order to evaluate the effect of the factors (can volume, time of sampling, preservative treatment) and their interactions on the distributions of parameters analyzed by chemical, physical, and microbiological analysis. Since results of the full factorial model did not show a significant effect (*p* > 0.05) on composition for the factor “can volume”, results were presented pooling samples based on the preservative treatment (NC, OAM and BSM). When the significance of the effect due to the interaction between “time of sampling” and “preservative treatment” was observed (*p* < 0.05), multiple comparisons were performed over group least square means by the Tukey’s HSD test. Then, a multiparametric approach was developed. The whole dataset was submitted to power transformation, chosen as a nonlinear conversion process that allowed the reduction of data heteroscedasticity. Subsequently, Pareto scaling was used to adjust significance of variables represented by large and small fold changes [21]. Finally, multilevel principal component analysis (multilevel-PCA) was applied as a multiparametric explorative tool, in order to reduce the dimensionality of the dataset while retaining the maximum of the original information and preserving the structure of the dataset (time and treatment). The final dataset consisted of a 48 (volatile organic compounds + pH + water activity + microbial counts) × 44 (samples) matrix. Univariate statistical analysis was performed by the JMP^®^ 15.2.0 tool of SAS (SAS Institute Inc., Cary, NC, USA). The multivariate analysis was performed by the mixOmics package of R and the PLS Toolbox of MATLAB.

## 3. Results and Discussion

### 3.1. PH, a_w_ (Water Activity), and Microbial Counts

Caviar analyzed in this study was associated with pH values ranging from 5.78 to 6.46 and a_w_ values ranging from 0.954 to 0.985, comparable to those reported by Bledsoe et al. [22] for fish roes coming from various species, included sturgeon. Since these values are known to be sufficiently high to support the growth of some microbial species, an initial low microbial load and a strict control of hygiene and sanitation measures are considered to have a primary importance in managing eventual contamination during the entire process of caviar production [23]. Actually, even when microbiologically sterile eggs are aseptically removed from fish, a secondary contamination during the production could occur, since this kind of product requires broad handling and multiple processing procedures [22,24]. Generally, the OAM and BSM series showed higher pH values for every sampling point when compared to salt samples. OAM and BSM samples ranged from 6.12 to 6.42, while the salt samples showed pH values always < 6, ranging from 5.84 to 5.97.

As reported in Table 2, the TVC recorded in this study at t0 showed very low values, always < 2 Log CFU/g in all the analyzed series, without any significant difference. Similar values recorded at t0 for the TVC in all three series suggested that, even if initial slightly different levels of microbial counts could be present because of individual fish characteristics, the processing conditions in the production plant were adequately standardized and effective in controlling the contamination. The microflora detected in this study was mainly composed of Bacilli and halophilic bacteria (data not reported), due to the natural microbial strains selection that occurred in the product related to the addition of salts.

Starting from t1, the TVC of the NC series showed a gradual increase, reaching average values up to 5.89, 5.90, and 6.89 Log CFU/g at t1, t2, and t3, respectively. The threshold limit of 6.0 Log CFU/g, frequently taken into consideration to designate the end of the shelf life of a fish product, was only recorded in the NC series at t2 and t3, corresponding to 8 and 14 months of ripening, respectively. In a previous study, Shin et al. [25] found that white sturgeon caviar treated with 3–3.75% of salt and stored at refrigeration temperatures (3–7 °C) reached the total microbial count of 6.0 Log CFU/g in a time range of only 5–10 days. This outcome evidenced that the strictly low temperature (−2 °C) sustained in the production plant for caviar analyzed in this study had a fundamental role in maintaining the food safety requirement for the NC product even in a longer term. Extended results for each replicate and additional data for Table 2 have been provided as supplementary materials (Table S1 of supplementary materials).

The results obtained in this study about caviar’s hygienic parameters can be considered positive, since the general picture regarding food safety of caviar and fish roes available in the literature is variable. As a matter of fact, a suitable hygienic level of processed fish roes from various species, including sturgeon, have been recently evidenced, with the limit of 6 Log CFU/g rarely exceeded in this kind of product [14,26]. On the contrary, other authors had previously detected higher microbial counts in both caviar [23] and other fish roes products [27], in the range 3–6.5 Log CFU/g.

The fact that in this study we found the highest microbial counts in NC samples supports the theory that salting in low concentration alone cannot be a sufficient step to inhibit completely the microbial growth in fish roes products if the shelf life is extended beyond 8 months [22]. Actually, in the OAM and BSM series, the TVC detected, very low since t0, did not show any relevant increase until the end of the study, showing a significant (*p* < 0.05) difference from the TVC detected in the NC at t1. At the end of the sampling time (t3), only one sample over the 4 in the BSM series showed the presence of microbial counts, with a value of 1.30 log CFU/g.

### 3.2. Volatile Profile

Results of the VOCs profile detected by HS-SPME-GC-MS at different times of ripening for each treatment are presented in Table 3 and Table 4.

Generally, we observed an enhancement of total VOCs amount during the sampling time in all the treatments tested. Aldehydes represented the first group for quantity, followed by alcohols (in all series), ketones (only in NC series), and acids (only OAM series). In all the series, sampling time matching 8 months of storage (t2) was detected as a “critical” time point, characterized by a massive enhancement of the signal provided by the VOCs detected, as a consequence of the development of volatile profile because of the ripening processes that occurred. This outcome is particularly interesting since the time corresponding to 6–8 months of ripening is considered by the producers as the optimal time range to obtain high-quality caviar for all the three treatments. Many compounds were found in all the treatments tested. Some of them were detected earlier at t0, then recorded in fluctuating amounts during the storage: 3-methylbutanal, benzaldehyde, octanal, nonanal, 2-phenylacetaldehyde, decanal, and nonanoic acid. Others were merely detected during the progression of the storage, as an outcome of the ripening processes: 2-methylbutanal, 3-methylsulfanylpropanal, heptanal, 2-hexenal, 2-nonenal, 4-methyl-1-heptanol, 6-methyl-1-octanol, and 1-phenylethanone. Most of these compounds are known to belong to the typical volatile profile of fish products, as all of them had been previously found as characteristic volatile in fish products [28,29,30,31,32], and also in ripened fish roes [18,33,34]. 2-methylbutanal, 3-methylbutanal, 2-phenylacetalhdehyde, and 3-methylsulfanylpropanal are Strecker aldehydes, known to be formed by the breakdown of amino acids: isoleucine for 2-methylbutanal, leucine for 3-methylbutanal and phenylalanine, and methionine for 2-phenyalcetaldehyde and 3-methylsulfanylpropanal [35,36,37,38]. 2-metylbutanal and 3-methylbutanal were found by Parlapani et al. [39] only in fish tissue models inoculated with bacterial strains, implying that these compounds were specific metabolites produced by the microbial spoilage. On the contrary, hexanal, heptanal, octanal, nonanal, and decanal were found by the authors also in non-inoculated samples, suggesting an enzyme-catabolic or oxidative origin. Actually, most of the saturated and unsaturated aldehydes ranging from 6 to 10 carbon atoms are secondary products of oxidation and enzyme-catabolism of polyunsaturated fatty acids (PUFA): heptanal, octanal, nonanal, decanal, 2-nonenal, and 2-hexenal, found in caviar in this study, are known to derive from the degradation of oleic, linoleic, linolenic, EPA, and DHA fatty acids peroxides [35,40]. During the storage, carbohydrates, amino acids, and lipids act as substrate for degradation processes promoted by microbial or oxidation spoilage. The rate of lipid oxidation is affected by several factors, including fatty acid composition and the unsaturation degree [35]. White sturgeon caviar contains 40% of PUFA over the total of fatty acids, with half of them being represented by n3-series FA [41]. For this reason, it can be deduced that the high unsaturation degree, associated, in many samples, with the presence of microbial strains, could have promoted the formation of secondary oxidation products with volatile properties, and probably had an impact on caviar flavor [35].

Many differences were detected among the three treatments tested. Samples treated with only sodium chloride (NC series) were those represented by the highest amounts of VOCs, associated to the highest increase until the final sampling time as well. Particularly, in the NC series we detected a substantial presence of alcohols and ketones (Table 4), distinctive specifically for this treatment. According to Olafsdottir et al. [42] and Jonsdottir et al. [43], who studied microbial metabolites in chilled fish, the formation of ketones in high levels, mainly represented by 3-hydroxy-2-butanone (also known as acetoin), can be associated to the active growth of specific spoilage organisms (SSO). Actually, acetoin is a microbial derived compound that can be formed from carbohydrate sources (via pyruvate and diacetyl), generally found in higher amounts if compared to lipid-derived ketones [44]. In this study, 3-hydroxy-2-butanone was found exclusively in NC samples, starting from t2 (6.55 Log peak area). The same was observed for 3-methyl-1-butanol, detected only in NC samples and significantly increasing (*p* < 0.01) from t2 (2.98 Log peak area) to t3 (6.26 Log peak area). Even 3-methyl-1-butanol has been previously noticed to increase by authors who investigated the volatile profile of fish products and fish roes during cold storage [29,32,33,45]. Particularly, Miller et al. [46] identified the presence of 3-methyl-1-butanol in sterile fish muscle inoculated with spoilage bacteria strains and incubated for 7 days, thus advising the formation of this alcohol by microbial spoilage. For this reason, 3-hydroxy-2-butanone and 3-methyl-1-butanol have been suggested as early indicators of spoilage in fish products. According to this, we found that NC samples were associated with the highest values of TVC detected, even closer to the critical value of 6 Log UFC/g (Table 2). As a matter of fact, even during low temperature storage, both enzymatic and non-enzymatic changes in fish tissues persist, and, even if in slower rates, they can still support microbial growth and metabolism [47]. Moreover, it is known that the use of no additive other than sodium chloride (in low concentrations) makes caviar suitable for consumption only in a relatively short time term (up to 6–8 months) [9] and with a storage temperature close to 0 °C. NaCl is known to have the power to reduce the autocatalytic activity in food products, but, when it is used as unique method of preservation without further processing, complete bacterial protection is not provided, because halophilic microorganisms can cause spoilage [35]. Thus, in such concentrations (<4%), it seemed to be not enough to sustain a strong antimicrobial activity alone.

The opposite condition was observed in the OAM series, which was represented by the lowest amount of VOCs, and showed a slower and more gradual increase throughout the ripening time. Actually, many compounds representative in NC and BSM samples were undetected in the OAM series. An example is represented by the unsaturated aldehydes 2-heptenal and 4-heptenal. 4-heptenal is an n3-series PUFA oxidation product previously found in other fish products [29,43,48] and ripened fish roes [33,34], well known to contribute to the expression of the overall cold stored and “fishy” flavor [49,50]. The absence of this compound in the OAM caviar samples suggests that oxidation phenomena occurred to a lesser degree in this series, probably because of the combination of the strongly controlled storage conditions and the efficiency of the preservative added, containing isoascorbic acid as an antioxidant. In a similar way, other compounds were found in the OAM series arising later during the storage time if compared to salt and BSM series. 1-penten-3-ol and 2-ethyl-1-hexanol were detected at t1 in NC and BSM while they appeared only at t2 in OAM samples. Similarly, 2,4-heptadienal, 2-octenal, and 1-octen-3-ol arose in NC and BSM samples at t2, but in OAM samples only at the end of the storage (t3) and in lower concentrations. These compounds are known to be related to the oxidation processes that occurs toward long-chain PUFA, and they have been found in the volatile profile of several fish products, also during cold storage [28,29,30,31,32,51,52] and also in ripened fish roes [33,34]. 1-penten-3-ol and 2,4-heptadienal are listed among the main products of n3-series PUFA autoxidation [40]; similarly, 1-octen-3-ol is known to be a degradation product of n6-series PUFA hydro peroxides (mainly, linoleic and arachidonic acid) [53]. Moreover, 2,4-heptadienal and 1-penten-3-ol have been evidenced as markers for early oxidation in n3-PUFA-enriched fish products, contributing to the sensorial property of rancidity and representing important contributors to off-flavors. Since some authors suggested that 1-penten-3-ol can be produced by microorganisms arousing amino acids and lipids degradation [46,54], it has been pointed to as an indicator of microbial spoilage as well. Actually, in papers published in 2008 and 2009, Iglesias et al. [28,55] showed that the presence of 1-penten-3-ol and 1-octen-3-ol in fish tissues was strongly correlated with the extent of fish oxidation phenomena occurring during the storage. Furthermore, in this study, the alcohol 2-ethyl-1-hexanol was found with a decreasing trend (between t1 and t2 in NC samples, *p* < 0.01) or completely disappearing (in BSM and OAM at t2 and t3, respectively) during the storage of caviar samples, according to what was previously found in literature [32,45]. 2-ethyl-1-hexanol is known to be formed during the storage of fish products in vacuum conservation conditions and at low temperatures [44,45]. Parlapani et al. [39] found this alcohol in both microbial-inoculated and non-inoculated fish tissues, suggesting that it could be a product of both non-microbial and microbial activity, but they detected significant higher amounts of this alcohol in microbial spoiled samples. According to this, we found the highest amount of 2-ethyl-1-hexanol in NC and BSM samples at t1 (5.87 and 5.70 Log peak area), while in the OAM samples, it was found only at t2 (5.31 Log peak area). The fact that 4-heptenal, 2,4-heptadienal, 1-penten-3-ol, 1-octen-3-ol, and 2-ethyl-1-hexanol were belatedly detected in the OAM series again agreed to the delayed process of oxidation hypothesized for caviar treated with this preservative.

**Figure 1:**
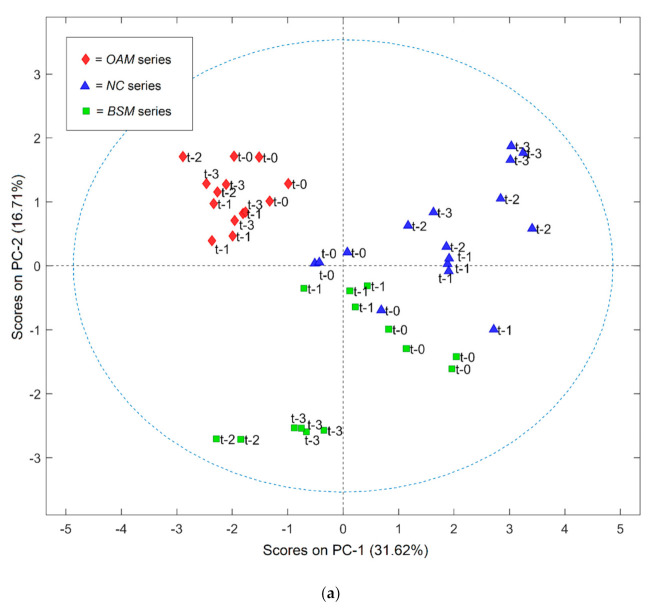

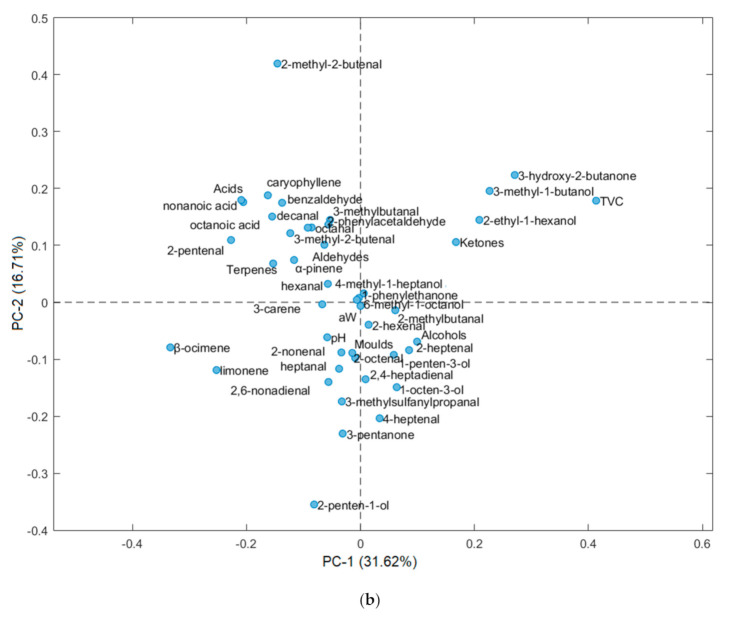
(**a**) Score plot of caviar samples analyzed in the study obtained by a multilevel principal component analysis (multilevel-PCA). t-0 = unripened roes, t-1 = 4 months of caviar ripening, t-2 = 8 months of caviar ripening, t-3 = 14 months of caviar ripening. (**b**) Loading plot of parameters measured in analyzed caviar samples obtained by multilevel-PCA. Variables associated with higher loadings on the 1st or the 2nd principal component (PC-1 and PC-2 on the x and the y axis, respectively) are related to a higher influence on variability recorded among samples in the multiple level data matrix.

In Figure 1a,b, the score plot (a) and the loading plot (b) obtained after the employment of a multilevel principal component analysis on the whole dataset (VOCs, pH, a_w_, microbial enumerable counts) are reported.

Changes in caviar chemical and microbiological profile occurred during the ripening time, and a tendency to form clusters among samples belonging to the same treatment can likely be observed in the multilevel-PCA score plot (Figure 1a). For all three series (NC, OAM, and BSM), an evolution from t0 and t3 was observed. Generally, the intensity of the signals recorded for volatile organic compounds’ peaks detected by chromatographic analysis showed an increase in all three series of samples from t0 to t3. This phenomenon could be linked to the proteolysis and hydrolysis phenomenon occurring toward polysaccharides and lipids during the first months of caviar aging, as previously indicated in the literature [8,15]. However, the evolution of the whole profile of parameters measured in samples was related to the increase of different compounds for each group, and, thus, lead to the distribution of scores in different quarters of the PC space. Particularly, the NC samples clearly showed an opposite trend in comparison to the other two treatments. During the sampling time, from t0 to t3, NC samples noticeably moved toward the area of the space related to the higher loadings of many compounds indicative of the occurrence of ripening and spoilage processes: 3-hydroxybutan-2-one, 3-methylbutan-1-ol, and 2-hethylhexan-1-ol. Similarly, the NC series was positively related on both PC-1 and PC-2 to the amount of viable counts detected (TVC, total viable count), showing a clear increase from t0 to t3. At t3, 3 samples out of 4 of the NC series showed the presence of microbial counts, reaching an average value of 6.89 log CFU/g. Thus, we evidenced that the NC samples at t3 showed a higher correlation with the values of all the parameters identified as markers of microbial spoilage. This outcome agreed with the fact that 14 months of storage are considered far and away the optimal ripening time for salted-only caviar from producers, usually indicated as 6–8 months maximum.

The microbial counts in this study were detected at different concentrations in the three series, according to the antimicrobial effectiveness of the preservatives employed. For the OAM series, no counts were evidenced at the end of the sampling time (t3). According to this, the OAM samples (the red diamonds in Figure 1b) showed the lowest dispersion from t0 and t3 and the highest tendency to form a condensed cluster characterized by scores inversely associated with the markers of spoilage processes. In the multilevel-PCA plot, this series was positively related on the PC-1 to the loadings for many compounds, such as terpenes and organic acids, which have not been associated with the spoilage of the product.

Finally, we observed an intermediate trend for the BSM series. A clear evolution was evidenced between t1 and t2 (4 and 8 months of ripening, respectively), with BSM moving toward the lower quarters of the PC space, positively related on the PC-2 with the loadings of many compounds, such as aldehydes, ketones, and alcohols, particularly 2-penten-1-ol. Then, the profile of the BSM series showed a smaller changes between t2 and t3. Actually, the time range t1–t2 was the one in which the most interesting changes on the volatile and microbial pattern was evidenced in all samples analyzed. In this time range, enzymatic, oxidative, and microbial activities took place in samples from all the series, leading to the formation of several chemical compounds responsible for the fishy flavor of caviar (2-penten-1-ol, 3-pentanone, 4-heptenal), but at a different rate in the different treatments. These outcomes appear interesting since actually, according to the producers, the sampling time t2 (corresponding to 8 months of ripening) is the only storage time that sustains the consumption of high-quality caviar for all the treatments tested.

## 4. Conclusions

The results obtained in this study evidenced interesting differences among caviar samples treated with different preservatives during a storage time of 14 months. The differences were particularly highlighted when combining all the parameters measured (physical, chemical, and microbiological) in a multivariate approach. The microbial counts (measured as total viable count, TVC) detected in samples analyzed in the present study generally evidenced an adequate management of samples processed in the production plant, leading to a good control of secondary contamination occurrence. However, the BSM (borax and salt mixture) and OAM (organic acid mixture) treatments were more effective in protecting the samples from the growth of microbial species in the long term (over 8 months) when compared to the addition of sodium chloride only (NC series). Similarly, all samples showed an increase for the chromatographic area of chemical compounds related to the ripening processes occurring toward sturgeon eggs during the sampling time. However, NC samples were associated to a volatile profile enriched in compounds driven by the spoilage occurring during the ripening processes (mainly, ketones). On the contrary, the OAM series showed the lowest amounts for all group of VOCs derived from the spoilage processes, while the BSM series showed an intermediate profile, although one characterized by the total absence of ketones. The outcomes obtained in this study showed that the BSM and, mainly, the OAM series displayed the highest stability of the product in the long term, suggesting the possibility of extending caviar shelf-life to a longer storage time (up to 14 months) when using such preservative mixtures.

## Figures and Tables

**Table 1:** Samples collection scheme. Legend: NC, sodium chloride (NaCl); OAM, organic acid mixture; BSM: borax and salt mixture.

			March 2019 Raw Eggs	July 2019 4 Months	November 2019 8 Months	May 2020 14 Months
ID	Series	Can	t0	t1	t2	t3
1	*NC* 3.8%	500 g	*n* = 2	*n* = 2	*n* = 2	*n* = 2
2						
3		1800 g	*n* = 2	*n* = 2	*n* = 2	*n* = 2
4						
1	*OAM* 4%	500 g	*n* = 2	*n* = 2	*n* = 1	*n* = 2
2						
3		1800 g	*n* = 2	*n* = 2	*n* = 1	*n* = 2
4						
1	*BSM* 3.8%	500 g	*n* = 2	*n* = 2	*n* = 1	*n* = 2
2						
3		1800 g	*n* = 2	*n* = 2	*n* = 1	*n* = 2
4						
N=			12	12	8	12

**Table 2:** pH, a_w_, and total viable count (TVC, expressed as Log CFU/g) detected in caviar under investigation during the ripening time. Data were expressed as mean ± SEM (standard error of the mean). For the TVC, the number of samples in which counts were detected is indicated in brackets. Legend: NC, sodium chloride (NaCl); OAM, organic acid mixture; BSM, borax and salt mixture. a_w_: water activity, TVC: total viable counts.

	pH	a_w_	Total Viable Count (Log CFU/g) and Countable
t0 = unripened roes			
NC	5.86 ± 0.02	0.30 ± 0.00	1.24 ± 0.17 b (2/4)
OAM	6.12 ± 0.09	0.30 ± 0.00	1.08 ± 0.08 b (4/4)
BSM	6.15 ± 0.01	0.30 ± 0.00	1.48 ± 0.20 b (4/4)
t1 = 4 months of ripening			
NC	5.97 ± 0.01	0.29 ± 0.00	5.89 ± 1.29 a (4/4)
OAM	6.28 ± 0.07	0.29 ± 0.00	1.20 ± 0.17 b (4/4)
BSM	6.42 ± 0.02	0.29 ± 0.00	1.50 ± 0.17 b (3/4)
t2 = 8 months of ripening			
NC	5.90 ± 0.02	0.29 ± 0.00	5.90 ± 1.20 a (4/4)
OAM	6.26 ± 0.01	0.29 ± 0.00	nd
BSM	6.31 ± 0.04	0.29 ± 0.00	nd
t3 = 14 months of ripening			
NC	5.84 ± 0.02	0.29 ± 0.00	6.89 ± 0.09 a (3/4)
OAM	6.12 ± 0.08	0.29 ± 0.0	nd
BSM	6.30 ± 0.03	0.29 ± 0.00	1.30 (1/4)

a,b = values associated to a different letter within the same column were significantly different (*p* < 0.05) when testing for the interaction effect of ripening time and preservative treatment over group least squares means (Tukey HSD multiple comparisons) in a multifactorial model; nd = not detected.

**Table 3:** Aldehydes detected in caviar during the ripening time for each treatment. Chromatographic areas are expressed in Log10. Data are presented as mean ($\bar{x})$ ± standard error of the mean (SEM). Legend: NC, sodium chloride (NaCl); OAM, organic acid mixture; BSM: borax and salt mixture.

	t0 = Unripened Roes			t1 = 4 Months of Ripening			t2 = 8 Months of Ripening			t3 = 14 Months of Ripening		
	NC N = 4	OAM N = 4	BSM N = 4	NC N = 4	OAM N = 4	BSM N = 4	NC N = 4	OAM N = 2	BSM N = 2	NC N = 4	OAM N = 4	BSM N = 4
	$\bar{x}$ ± SEM	$\bar{x}$ ± SEM	$\bar{x}$ ± SEM	$\bar{x}$ ± SEM	$\bar{x}$ ± SEM	$\bar{x}$ ± SEM	$\bar{x}$ ± SEM	$\bar{x}$ ± SEM	$\bar{x}$ ± SEM	$\bar{x}$ ± SEM	$\bar{x}$ ± SEM	$\bar{x}$ ± SEM
3-methyl butanal	5.21 ± 0.03 d	5.38 ± 0.04 cd	3.91 ± 0.28 e	5.51 ± 0.32 bcd	5.73 ± 0.10 cd	5.48 ± 0.08 bcd	6.36 ± 0.07 a	6.26 ± 0.04 a	6.03 ± 0.02 ab	6.36 ± 0.05 bcd	6.32 ± 0.06 a	5.96 ± 0.08 abc
2-methyl butanal	nd	nd	nd	4.98 ± 0.31 b	5.22 ± 0.09 b	4.99 ± 0.10 b	5.92 ± 0.06 a	nd	5.51 ± 0.00 ab	5.95 ± 0.07 a	5.91 ± 0.06 a	5.54 ± 0.09 ab
2-methyl, 2-butenal	nd	5.46 ± 0.09 b	nd	nd	4.89 ± 0.07 c	nd	nd	5.79 ± 0.02 a	nd	5.84 ± 0.16 a	5.53 ± 0.08 ab	nd
2-pentenal	nd	5.36 ± 0.05 ab	nd	nd	4.82 ± 0.04 c	nd	nd	5.49 ± 0.04 a	5.22 ± 0.03 ab	5.18 ± 0.08 bc	5.40 ± 0.14 ab	5.24 ± 0.09 ab
3-methyl, 2-butenal	nd	nd	nd	nd	nd	nd	nd	2.50 ± 1.77 ab	nd	nd	4.87 ± 0.07 a	nd
Hexanal	5.58 ± 0.26 def	5.78 ± 0.05 cdef	4.59 ± 0.29 g	5.43 ± 0.37 ef	5.37 ± 0.07 f	5.91 ± 0.10 cdef	6.14 ± 0.19 bcd	5.98 ± 0.06 bcde	6.76 ± 0.00 a	6.60 ± 0.10 ab	6.35 ± 0.17 abc	6.68 ± 0.08 ab
2-hexenal	nd	nd	nd	nd	nd	nd	4.99 ± 0.07 bc	4.55 ± 0.04 c	5.44 ± 0.08 a	5.30 ± 0.08 ab	4.92 ± 0.16 bc	5.28 ± 0.11 ab
4-heptenal	nd	nd	nd	nd	nd	nd	nd	nd	5.23 ± 0.02 a	4.99 ± 0.07 b	nd	5.20 ± 0.11 ab
Heptanal	nd	nd	nd	nd	nd	nd	nd	nd	5.44 ± 0.02 a	5.36 ± 0.08 ab	5.16 ± 0.14 b	5.39 ± 0.08 ab
3-methyltio, propanal	nd	nd	nd	4.88 ± 0.13 de	5.01 ± 0.15 cd	4.43 ± 0.10 e	5.71 ± 0.13 ab	5.87 ± 0.03 a	5.37 ± 0.04 bc	nd	nd	5.43 ± 0.08 bc
2-heptenal	nd	nd	nd	nd	nd	nd	nd	nd	nd	5.09 ± 0.08 a	nd	5.13 ± 0.09 a
Benzaldehyde	4.96 ± 0.23 cde	5.53 ± 0.05 abc	nd	4.39 ± 0.34 e	5.07 ± 0.06 bcde	4.67 ± 0.05 de	5.55 ± 0.06 abc	5.84 ± 0.04 a	5.58 ± 0.02 abc	5.34 ± 0.04 abcd	5.74 ± 0.06 ab	5.44 ± 0.08 abc
Octanal	5.04 ± 0.28 ab	5.18 ± 0.17 a	3.48 ± 0.30 d	4.16 ± 0.35 cd	4.58 ± 0.05 abc	4.36 ± 0.11 bc	4.99 ± 0.05 ab	4.97 ± 0.04 ab	5.20 ± 0.02 a	5.36 ± 0.06 a	5.27 ± 0.08 a	5.13 ± 0.07 ab
2,4-heptadienal	nd	nd	nd	nd	nd	nd	2.47 ± 1.43 b	nd	5.60 ± 0.03 a	5.30 ± 0.09 a	3.72 ± 1.25 ab	5.39 ± 0.14 a
Benzeneacetaldehyde	4.88 ± 0.30 c	5.36 ± 0.14 bc	3.95 ± 0.16 d	5.45 ± 0.34 bc	5.68 ± 0.16 abc	5.46 ± 0.06 bc	6.47 ± 0.10 a	6.33 ± 0.02 a	6.14 ± 0.02 ab	6.24 ± 0.09 ab	6.23 ± 0.07 ab	5.96 ± 0.07 ab
2-octenal	nd	nd	nd	nd	nd	nd	2.54 ± 1.47 b	nd	5.43 ± 0.04 a	5.28 ± 0.12 a	5.10 ± 0.19 a	5.29 ± 0.13 a
Nonanal	5.83 ± 0.27 ab	*	4.53 ± 0.29 d	5.19 ± 0.30 c	*	5.37 ± 0.05 bc	5.72 ± 0.05 abc	*	6.00 ± 0.06 a	6.10 ± 0.05 a	*	6.05 ± 0.05 a
2,6-nonadienal	nd	nd	nd	nd	nd	nd	nd	nd	5.25 ± 0.05 a	nd	nd	nd
2-Nonenal	nd	nd	nd	nd	nd	nd	nd	nd	4.87 ± 0.04 a	5.00 ± 0.05 a	4.93 ± 0.11 a	4.90 ± 0.06 a
Decanal	4.58 ± 0.24	4.83 ± 0.10	4.22 ± 0.17	4.15 ± 0.32	4.65 ± 0.03	4.53 ± 0.00	4.54 ± 0.08	4.91 ± 0.03	4.73 ± 0.04	5.07 ± 0.09	5.10 ± 0.08	4.96 ± 0.07
Sum of aldehydes	6.22 ± 0.22 c	6.35 ± 0.05 bc	5.03 ± 0.26 d	6.16 ± 0.31 c	6.28 ± 0.09 c	6.28 ± 0.05 c	6.99 ± 0.06 ab	6.88 ± 0.00 ab	7.09 ± 0.00 a	7.12 ± 0.05 a	6.98 ± 0.06 ab	7.02 ± 0.07 a

* = the chromatographic peak for nonanal in the OAM series was not possible to integrate because a coeluition occurred with a huge peak of sorbic acid (present in the mixture); nd = not detected; a–g = values associated to a different letter within the same row were significantly different (*p* < 0.05) when testing for the interaction effect of ripening time and preservative treatment over group least squares means (Tukey HSD multiple comparisons) in a multifactorial model.

**Table 4:** Alcohols, ketones and terpenes detected in caviar during the ripening time. Chromatographic areas are expressed in Log10. Data are presented as mean ($\bar{x}$) ± the standard error of the mean (SEM). Legend: NC, sodium chloride (NaCl); OAM, organic acid mixture; BSM: borax and salt mixture.

t0 = Unripened Roes				t1 = 4 Months of Ripening			t2 = 8 Months of Ripening			t3 = 14 Months of Ripening		
	NC N = 4	OAM N = 4	BSM N = 4	NC N = 4	OAM N = 4	BSM N = 4	NC N = 4	OAM N = 2	BSM N = 2	NC N = 4	OAM N = 4	BSM N = 4
	$\bar{x}$ ± SEM	$\bar{x}$ ± SEM	$\bar{x}$ ± SEM	$\bar{x}$ ± SEM	$\bar{x}$ ± SEM	$\bar{x}$ ± SEM	$\bar{x}$ ± SEM	$\bar{x}$ ± SEM	$\bar{x}$ ± SEM	$\bar{x}$ ± SEM	$\bar{x}$ ± SEM	$\bar{x}$ ± SEM
1-penten-3-ol	nd	nd	nd	4.75 ± 0.35 d	nd	5.25 ± 0.03 cd	5.53 ± 0.12 bc	5.08 ± 0.02 cd	6.10 ± 0.03 a	5.65 ± 0.09 bc	5.58 ± 0.16 bc	6.06 ± 0.05 ab
3-methyl,1-butanol	nd	nd	nd	nd	nd	nd	2.98 ± 1.72 b	nd	nd	6.26 ± 0.14 a	nd	nd
2-penten-1-ol	nd	nd	nd	nd	nd	nd	nd	nd	5.57 ± 0.02 a	nd	nd	5.49 ± 0.05 b
1-octen-3-ol	nd	nd	nd	4.52 ± 0.38 ab	nd	4.81 ± 0.15 ab	5.32 ± 0.02 bc	nd	5.98 ± 0.04 a	5.97 ± 0.11 a	5.62 ± 0.18 a	6.08 ± 0.10 a
2-ethyl, 1-hexanol	nd	nd	nd	5.70 ± 0.32 ab	nd	5.87 ± 0.12 a	4.85 ± 0.06 c	5.31 ± 0.02 ab	nd	5.18 ± 0.03 bc	nd	nd
4-methyl, 1-heptanol	nd	nd	nd	nd	nd	nd	nd	nd	nd	5.83 ± 0.26 a	5.73 ± 0.06 a	5.54 ± 0.10 a
6-methyl, 1-octanol	nd	nd	nd	nd	nd	nd	nd	nd	nd	5.41 ± 0.11	5.54 ± 0.04	5.35 ± 0.08
Sum of alcohols	nd	nd	nd	5.78 ± 0.33 cd	nd	6.01 ± 0.09 bcd	5.98 ± 0.09 bcd	5.51 ± 0.01 d	6.41 ± 0.03 ab	6.65 ± 0.13 a	6.25 ± 0.10 abc	6.52 ± 0.05 ab
												
3-pentanone	nd	nd	nd	nd	nd	nd	nd	nd	nd	nd	nd	5.06 ± 0.11
3-hydroxy, 2-butanone	nd	nd	nd	nd	nd	nd	6.55 ± 0.31	nd	nd	6.83 ± 0.19	nd	nd
Acetophenone	nd	nd	nd	nd	nd	nd	nd	nd	nd	4.97 ± 0.06 b	5.15 ± 0.04 a	4.99 ± 0.04 b
Sum of ketones	nd	nd	nd	nd	nd	nd	6.55 ± 0.31 a	nd	nd	6.84 ± 0.19 a	5.15 ± 0.04 b	5.35 ± 0.05 b
												
α-pinene	5.10 ± 0.29 abc	5.65 ± 0.10 a	4.71 ± 0.32 bc	4.63 ± 0.31 c	5.24 ± 0.09 abc	5.29 ± 0.07 abc	5.38 ± 0.12 abc	5.57 ± 0.08	5.66 ± 0.04 a	5.35 ± 0.09 abc	5.59 ± 0.09 ab	5.58 ± 0.07 ab
3-carene	nd	nd	nd	3.72 ± 0.32	4.43 ± 0.06	4.27 ± 0.01	4.64 ± 0.01	4.73 ± 0.01	4.76 ± 0.03	5.13 ± 0.05	5.13 ± 0.04	5.06 ± 0.05
Limonene	4.82 ± 0.04 b	5.33 ± 0.07 a	4.23 ± 0.25 c	nd	4.93 ± 0.20 abc	nd	nd	nd	5.20 ± 0.04 a	nd	5.28 ± 0.06 a	5.21 ± 0.05 ab
Β-ocimene	4.68 ± 0.06 c	5.51 ± 0.09 a	4.11 ± 0.34 d	nd	5.30 ± 0.12 ab	4.88 ± 0.10 bc	nd	5.42 ± 0.12 a	5.29 ± 0.04 ab	nd	5.41 ± 0.07 a	5.32 ± 0.07 ab
Caryophyllene	nd	5.05 ± 0.05	nd	nd	4.47 ± 0.09	3.79 ± 0.00	nd	4.23 ± 0.45	nd	nd	4.81 ± 0.08	nd
Sum of terpenes	5.46 ± 0.12 ab	6.03 ± 0.08 a	4.92 ± 0.30 bc	4.68 ± 0.31 c	5.75 ± 0.06 a	5.47 ± 0.07 ab	5.41 ± 0.13 abc	5.86 ± 0.10 a	5.94 ± 0.04 a	5.57 ± 0.06 ab	6.01 ± 0.06 a	5.94 ± 0.06 a

nd = not detected; a–d = values associated to a different letter within the same row were significantly different (*p* < 0.05) when testing for the interaction effect of ripening time and preservative treatment over group least squares means (Tukey HSD multiple comparisons) in a multifactorial model.

## Supplementary Materials

The following are available online at https://www.mdpi.com/article/10.3390/foods10040850/s1, Table S1: Extended with replicates and additional data for Table 2 in main text.
