# Supplementary material for: Evolution of Food Safety Features and Volatile Profile in White Sturgeon Caviar Treated with Different Formulations of Salt and Preservatives during a Long-Term Storage Time
Source: Foods. 2021 Apr 14;10(4):850. doi: 10.3390/foods10040850 (PMC8070692; doi:10.3390/foods10040850)
Supplement: Supplementary file 1 [file foods-10-00850-s001.pdf]

Table 2. - extended with replicates and additional data.

t0= unripened roe

	NC						*OAM*						*BSM*					
**Sample no**	1	2	3	4	mean	countable	1	2	3	4	mean	countable	1	2	3	4	mean	Countable
**pH**	5.84	5.88	5.84	5.88	5.86		6.13	6.23	6.3	5.83	6.12		6.17	6.12	6.16	6.14	6.12	
**aw**	0.974	0.977	0.974	0.982	0.977		0.972	0.972	0.969	0.985	0.975		0.978	0.975	0.975	0.976	0.975	
**TVC**	<1	1	1.48	<1	1.24	2/4	1	1	1.30	1	1.08	4/4	1	1.30	1.78	1.85	1.48	4/4
**Halophiles**	<1	<1	<1	1	1	1/4	1.30	<1	1.30	<1	1.30	2/4	<1	<1	<1	<1	<1	4/4
Bacilli	1.30	1.30	1	<1	1.20	3/4	<1	1	<1	<1	1	1/4	1	<1	<1	1.6	1.30	2/4
**Moulds**	<1	<1	<1	<1	<1	4/4	<1	<1	1	<1	1	1/4	<1	<1	<1	<1	<1	4/4

t1= 4 months of caviar ripening

	NC						*OAM*						*BSM*					
**Sample no**	1	2	3	4	mean	countable	1	2	3	4	mean	countable	1	2	3	4	mean	Countable
**pH**	5.96	5.96	6.01	5.95	5.97		6.33	6.35	6.42	6.03	6.28		6.44	6.41	6.37	6.46	6.42	
**aw**	0.966	0.963	0.962	0.964	0.964		0.96	0.97	0.954	0.961	0.961		0.97	0.968	0.961	0.963	0.966	
**TVC**	5.81	6.02	5.21	6.51	5.89	4/4	1.60	1.00	<1	1.00	1.20	3/4	1.30	1.30	<1	1.90	1.50	¾
**Halophiles**	4.48	4.30	4.48	4.60	4.46	4/4	1.00	<1	<1	<1	1	1/4	1	1	1.30	1.30	1.15	4/4
Bacilli	6.48	6.30	5.47	6.30	6.14	4/4	3.30	1.30	1.48	1.00	1.77	4/4	<1	<1	<1	2.08	2.08	¼
**Moulds**	<1	<1	<1	<1	<1	4/4	<1	<1	<1	<1	<1	4/4	<1	<1	<1	<1	<1	4/4

t2= 8 months of caviar ripening

	NC						*OAM*						*BSM*					
**Sample no**	1	2	3	4	mean	countable	1		3		mean	countable	1			4	mean	countable
**pH**	5.9	5.94	5.93	5.83	5.90		6.24		6.27		6.26		6.34			6.27	6.31	
**aw**	0.959	0.961	0.96	0.963	0.961		0.962		0.963		0.963		0.97			0.966	0.968	
**TVC**	2	6.94	6.65	6.78	4.59	4/4	<1		<1		<1	2/2	<1			<1	<1	2/2
**Halophiles**	<1	6.72	<1	<1	6.72	1/4	<1		<1		<1	2/2	3.34			<1	3.34	1/2
Bacilli	2.30	<1	6.64	6.95	5.30	3/4	<1		<1		<1	2/2	<1			<1	<1	2/2
**Moulds**	<1	1.60	<1	<1	1.60	1/4	<1		<1		<1	2/2	<1			<1	<1	2/2

t3= 14 months of caviar ripening

	NC						*OAM*						*BSM*					
**Sample no**	1	2	3	4	mean	countable	1	2	3	4	mean	countable	1	2	3	4	mean	countable
**pH**	5.87	5.85	5.87	5.78	5.84		6.16	6.23	6.25	5.85	6.12		6.38	6.31	6.2	6.3	6.30	
**aw**	0.96	0.955	0.956	0.957	0.957		0.957	0.956	0.954	0.954	0.955		0.966	0.959	0.963	0.958	0.962	
**TVC**	<1	7	6.68	7	6.89	3/4	<1	<1	<1	<1	<1	4/4	<1	<1	1.3	<1	1.3	1/4
**Halophiles**	1.7	7	6.83	6.3	5.46	4/4	<1	1	<1	<1	1	1/4	1	1	1	1.3	1.08	4/4
Bacilli	<1	<1	<1	<1	<1	4/4	<1	<1	1.48	1.7	1.59	2/4	1.3	<1	<1	<1	1.3	1/4
**Moulds**	1.3	<1	<1	1.48	1.39	2/4	<1	1	1	2.18	1.39	3/4	1.78	1.6	2.45	1	1.71	4/4

Materials and methods of data not presented in the manuscript.

Moulds were counted on Sabouraud Agar (ISO 21527-1:2008, [1]).

presumptive Bacillus cereus was enumerated onto PEMBA (ISO 7932:2004 [2].

halophilic bacteria were enumerated onto Tryptic Soy Agar supplemented with NaCl (30 g/L) then incubated at 30°C for 48h.

All the media were supplied by Scharlab (Barcelona, E).

Reference

1. International Organization for Standardization (ISO) Microbiology of Food and Animal Feeding Stuffs—Horizontal Methods for the Enumeration of Yeasts and Moulds—Part 1: Colony Count Technique in Products with Water Activity Greater than 0, 95; ISO 21527-1:2008 2008.
2. International Organization for Standardization (ISO) Microbiology of food and animal feeding stuffs — Horizontal method for the enumeration of presumptive Bacillus cereus — Colony-count technique at 30 degrees C. ISO 7932:2004 2004.
